# Editorial Notes

**Published:** 1919

**Authors:** 


### Editorial Notes.

**History of the Bristol Royal Infirmary.**

We have received from Dr. A. Lendon an interesting communication suggested by the late Dr. Munro Smith's *History of the Bristol Royal Infirmary,* which we print below *in extenso.* Dr. Lendon, who will be remembered by several of the senior members of the Royal Infirmary staff, has attained a distinguished position in the medical profession of Australia.

"The *History of the Bristol Royal Infirmary* by the late Dr. G. Munro Smith possesses an interest for at least four persons in this city, three of whom are medical men. The layman is the Hon. Sir John Langdon Bonython, C.M.G., a collateral descendant of that Dr. John Bonython who was one of the principal founders of the Infirmary. Sir Langdon, who came to South Australia as a child, has worked his way up by sheer industry and talent from a humble position to become the proprietor of the chief daily newspaper of this State. His residences are named 'Carclew' and 'Carminow,' after the places in Cornwall with which his ancestors were connected, and he claims to be the head of the well-known family which formerly flourished in the 'Delectable Duchy.'

"Recently the Medical Superintendent of the Adelaide Hospital was Dr. Charleton Yeatman, a graduate of the Adelaide University, and now Lieut.-Col. and C.O. of the Australian Hospital at Harefield. In the *History* the name of Charleton Yeatman occurs as that of the Resident Apothecary for a few months in 1783, whilst a brother of his, Morgan Yeatman, was Surgeon to the Infirmary at the same time. Dr. Charleton Yeatman's father (Dı. J. W. Yeatman) has been practising at Auburn, a township some forty miles from Adelaide, for thirty-six years. He tells us that the Apothecary (C.Y.) of 1783 was his great-grandfather, and that as his parents were second cousins of the same name he is therefore descended from the Surgeon as well as from the Apothecary in a direct line.

"The longest registered practitioner remaining on the South Australian Roll is John Sprod, M.R.C.S.: he is a native of South Australia, and he went to England in 1868 for his medical education. He was a surgical pupil attached to Mr. Leonard Crosby for two years, and was clinical clerk to Dr. Farebrother. He qualified in 1873, and returned next year to Adelaide.

"The fourth person is myself. I was House Surgeon—there was then only one—from 1879 to 1883. My resident colleagues at first were the Medical Superintendent, J. H. Lee Macintire (his name is incorrectly spelt on p. 359), and the House Physician, Dr. James Scott; the latter shortly afterwards resigned, and Macintire, myself, and Dr. C. H. Watson, were joint residents, till Watson left at Christmas, 1882, and I myself at Easter in the following year. J. Paul Bush succeeded Watson and then took my place.

"On page 355 there is, if my recollection of an event which took place nearly four decades ago be accurate, a slight error. The Lister demonstration took place, I think, in a large underground room of the central block, presumably the Out-Patient Waiting Room and not in the Museum. I may be wrong also in another respect, but I fancy the case was one of a large 'cold' abscess of the upper arm, but this is a trifling matter. I assisted Mr. Lister: we had reserved for his choice other cases, such as tumour and an amputation, but he preferred to demonstrate his method on this simple class of case, for it was in such that he won his early triumphs over sepsis.

"I perhaps ought to apologise for such a lengthy epistle, but I thought your readers would like to know that the *History* was appreciated at the Antipodes and that 'Caelum, non animum, mutant qui trans mare currunt.'"

**Postgraduate Courses.**

In "Medical Notes" will be found a notice which has been widely circulated amongst the medical men of Bristol and the neighbourhood, and, we are glad to say, has met with an excellent response. The weekly demonstrations have been very well attended, and many busy practitioners from distant country districts have been present at the meetings. In addition quite a number of medical men have taken posts at one or other of the Institutions as Clinical Assistants.

We should like to draw the attention of those taking the course to the fact that they may attend clinical rounds in the wards and visit the out-patient and special departments whenever they choose to avail themselves of the opportunity. They are entitled to a total number of attendances equivalent to one month's study, and such attendances may be distributed over any part of the present course.

We wish this new departure every success, and trust that it may undergo a great development in future years.

**The Case of Dr. J. H. Bell.**

In our last issue attention was directed to the case of Dr. John Henderson Bell, of Chelsea, who was at that time in prison, sentenced under the Defence of the Realm Act on evidence so flimsy that we could not escape the conviction that it was a miscarriage of justice. An appeal to the Home Secretary for re-trial had been refused on the ground that no fresh evidence had been adduced since the conviction, but as no fresh evidence was possible, if one could dignify the grounds of the doctor's conviction by the term evidence, one must be thankful that the consideration by the General Medical Council of the question of striking off the prisoner's name from the Register afforded this judicial body the opportunity of finding that Dr. J. H. Bell had not been guilty of "infamous conduct in a professional sense." Consequently he was set free. The trial scene in *Alice in Wonderland* may be very funny in real life but was a dignified procedure compared with this case in British law. One must hope that time and a continuance of his useful, upright life will enable Dr. Henderson Bell to forgive and to forget this painful episode in which, among all those concerned, he was the most creditable figure.

**The Late Sir Barclay Josiah Baron.**

THE universal expressions of deep regret which the news of the untimely death of this distinguished colleague called forth in this city is most fully shared by the Editorial Staff of this Journal, for which he did good work in years gone by. We confess to a sense of jealousy in that the claims of civic life had made such inroads on his time and interest during the latter years of his busy life ; but he proved himself a man of parts in so many directions and undoubtedly was of very great public service during the two years he filled the office of Lord Mayor during the earlier years of the Great War with dignity and in a manner that few of his fellow-citizens could have equalled and none could have excelled. We had looked forward to his enjoying his well-won honours for many years and to his resuming more fully his work as a laryngologist.

**Closing of the 2nd Southern General Hospital.** WHILE it is impossible to make an adequate reference here to the work accomplished by the 2nd Southern General Hospital during the War, the closing down of the great Military Hospital organised in our midst demands a note and calls forth the admiration for the sustained effort of those whose work did such yeoman service in the time of national need and peril.

The new King Edward VII. wing of the Bristol Royal Infirmary had been offered for use as a Military Hospital in the event of war long before the Great War was dreamt of, and immediately war was declared Lieut.-Colonel Paul Bush, C.M.G., as he then was, called his *à la suite* staff together and under the Director-General organised for the Royal Army Medical Corps the 2nd Southern General Hospital, with the Royal Infirmary block as the organising centre, till with its various sections and auxiliary hospitals about 5,800 beds were provided, and during the war very little short of 100,000 patients were dealt with. It had been in full working order for three years under the administration of Lieut.-Colonel Bush, when he was sent to France to organise a hospital at the base as Colonel Bush, and was succeeded as O.C. of 2nd Southern General Hospital by Lieut.-Colonel Prowse. On another occasion we hope to record in more detail the great work that was done in Bristol and throughout a widely extending neighbourhood by the 2nd Southern General Hospital.

**War Honours.** TO our many colleagues and the men of the Bristol School who have received honourable distinctions for their war services since our last issue we offer warm congratulations. On another page we give a list of their names and honours, but we may be excused in specially mentioning here the granting of a C.B.E. to Colonel Paul Bush, C.M.G., A.D.M.S., Colonel James Swain, C.B., Colonel H. Thurston, C.B., C.M.G., Colonel P. G. Stock, D.S.O., Lt.-Colonel J. H. Parsons, and Capt. Hele.

**Hospital Amalgamation.**

It has often been said that the chief factor in modern social and political developments has been the immense improvement which has taken place in means of communication and transport. The result of bringing distant parts of the world so close together has been, in all spheres of activity, an enlargement of the effective unit of competition. Towns which were formerly rivals have to combine against distant competitors; large businesses amalgamate; where formerly small states competed with one another international leagues are instituted to carry out wider programmes of rivalry.

To turn from international to domestic matters, it has become apparent to many both inside and outside the profession that in Bristol there is no longer room for rivalry between the various hospital institutions, and that if the city is to maintain its position as a centre for the advancement and teaching of medical science, it must be ready to compete as a combined unit with the other large cities of the kingdom, and of the empire. These ideas have begun to take practical form. A committee consisting of representatives of the governing bodies and professional staffs of the leading hospitals of the city has been formed and is considering a scheme for amalgamation of both the administrative and medical work of these institutions. Additional stimulus has been given to the movement by the action of the Board of Education, which is prepared to assist medical education and research in a very substantial way by means of grants from national revenue. To obtain the full benefit of this scheme it is, in the opinion of the medical staffs, essential that their resources should be combined ; and this, we understand, is also the view taken by the Board of Education. From the financial and administrative point of view there are obvious advantages in amalgamation, as it will avoid much duplication of apparatus and overlapping of interests, while it will enable all purchases to be made in larger quantity and at a cheaper rate. There are, of course, difficulties in detail, but none are insuperable, and it is to be hoped and expected that before long Bristol will possess a centralised and amalgamated system of medical institutions which will enable it to more than hold its own among the great centres of medical activity in the kingdom.

